# Efficacy of the adjunctive use of photobiomodulation therapy in olfactory disorders in post-COVID-19 patients: A randomized controlled trial

**DOI:** 10.1016/j.bjorl.2025.101583

**Published:** 2025-03-26

**Authors:** Patricia Costa Oliveira, Luisa Oliveira Correia, Natalia Medeiros Dias Lopes, Gabriel Rodrigues Suassuna, Richard L. Doty, Fabio de Rezende Pinna, Richard Louis Voegels, Marco Aurelio Fornazieri

**Affiliations:** a Universidade Estadual de Londrina, Londrina, PR, Brazil; b Pontifícia Universidade Católica do Paraná, Londrina, PR, Brazil; c GEM, Londrina, PR, Brazil; d Smell and Taste Center, Department of Otorhinolaryngology: Head and Neck Surgery, Perelman School of Medicine, University of Pennsylvania, Philadelphia, Pennsylvania, United States; e Universidade de São Paulo, São Paulo, SP, Brazil

**Keywords:** Olfactory disorders, Smell, COVID-19, Photobiomodulation, Laser therapy

## Abstract

**Objectives:**

Photobiomodulation Therapy (PBMT) is a non-invasive treatment that uses photons from the red to infrared spectrum to modulate cellular processes with anti-inflammatory and regenerative properties. This study aimed to evaluate the use of PBMT applied intranasally in patients with an impaired sense of smell after COVID-19. Methods: This proof-of-concept study was a randomized, placebo-controlled clinical trial that recruited 81 patients with olfactory disorders after SARS-CoV-2 infection (1–12 months), randomly assigned to 3 groups: controls, red light and infrared light exposure groups. PBMT was applied twice a week for five weeks, associated with prednisolone 40 mg for five days and olfactory training for 90 days from the first day of laser application. UPSIT® and subjective chemosensory scores were the outcomes collected before the first session and three months thereafter.

**Results:**

Compared to controls, patients undergoing infrared PBMT showed more improvement in UPSIT® scores (+4.6 points, 95% CI: 1.5–7.8, *p* =  0.004) and a tendency towards reporting a better subjective smell score at the three-month follow-up. Response rates were 26.1% (95% CI: 6.7–45.5), 43.5% (95% CI: 21.6–65.4), and 68% (95% CI: 48.3–87.7) in the control, red and infrared groups, respectively. No major adverse events were reported.

**Conclusion:**

Therapy with PBMT in the infrared frequency appears to be a safe option in treating post-COVID olfactory disorders when combined with a five-day use of systemic corticosteroid and 90 days of olfactory training.

**Level of evidence:**

2.

## Highlights

• PBMT with steroids and training improved post-COVID olfaction, especially infrared.
• Infrared had a 68% response rate, surpassing red light (43.5%) and control (26.1%).
• PBMT with steroids and olfactory training was safe for treating smell disorders.

## Introduction

COVID-19 is an infectious viral disease with a high potential for propagation and pathogenicity that has a varied clinical picture, with olfactory disturbances and taste loss being one of the leading and most frequently reported clinical manifestations during the first two years of the pandemic.^1^ It has been shown that SARS-CoV-2 infects cells of the olfactory epithelium and triggers an inflammatory process that releases large amounts of cytokines and neurotoxic compounds, causing vascular hypoperfusion. These mechanisms lead to decreased expression of odor receptors and olfactory neuron death.^1^, ^2^ The patients’ olfactory recovery after COVID-19 typically takes an average of 15-days.^3^, ^4^ However, more than 20% of patients report some degree of olfactory dysfunction even two years after of the infection.^5^, ^6^, ^7^, ^8^

Among current treatments for olfactory dysfunction after COVID-19 are olfactory training, topical intranasal medications, and oral anti-inflammatory- neuroprotective agents.^9^, ^10^ Olfactory training is one of the main forms of treatment.^11^ Besides this, a variety of vitamins, minerals and nutritional supplements have been proposed to be of benefit - either taken as an oral supplement or, in some instances, used intranasally (such as intranasal vitamin A drops).^12^ Glutamate antagonists and xanthine derivatives are used occasionally in the treatment of post-viral olfactory dysfunction and may therefore be assessed in relation to COVID-19. Trials of acupuncture have also taken place.^12^ Other therapies used in non-COVID-19 olfactory dysfunction, such as theophylline, vitamin A, omega-3, or zinc, have been investigated but lack compelling evidence in favor of their use.^13^ Unfortunately, their effects rarely reach normosmic levels.^14^, ^15^, ^16^ This study evaluated the effectiveness of the application of Photobiomodulation Therapy (PBMT) in olfactory disorders of post-COVID-19 patients as an adjunct to olfactory training and systemic corticosteroids. The clinical application of PBMT has been increasing in many areas of medicine, such as wound healing, inflammatory disorders, and autoimmune and neurodegenerative diseases.^17^, ^20^, ^21^

PBMT uses a laser (“light, amplification, stimulated, emission, radiation”) to trigger photophysical and photochemical events in biological tissues and cells to an emission of photons from the red to infrared spectrum, thereby modulating cellular processes leading to therapeutic benefits. The mechanism of action of PBMT involves the absorption of photons within intracellular mitochondria by cytochrome-C-oxidase, producing Adenosine Triphosphate (ATP), which drives cellular metabolism to generate cell proliferation, angiogenesis, and decreased cytokine release.^22^, ^23^, ^24^ Through this, the laser's anti-inflammatory, analgesic, and biomodulation effect on the body is observed.^25^, ^26^, ^27^, ^28^

Unlike conventional pharmacology, which affects multiple body receptors,^18^, ^19^, ^26^ This randomized placebo-COntrolled clinical trial aimed to evaluate the effectiveness of the adjunctive use of photobiomodulation therapy on olfactory disorders in post-COVID-19 patients.

## Methods

This single-blinded, randomized, controlled clinical trial was approved by the Research Ethics Committee of the State University of Londrina under approval No. 4.150.690 in Londrina, Parana, Brazil. It was registered at ensaiosclinicos.gov.br, a registry of clinical trials, under nº RBR-2mvznz3. Following signed consent, the study protocol was explained in detail to each potential participant and followed Resolution nº 466/2012 of the National Health Council and Declaration of Helsinki.

### Patients

**Fig. 1:**
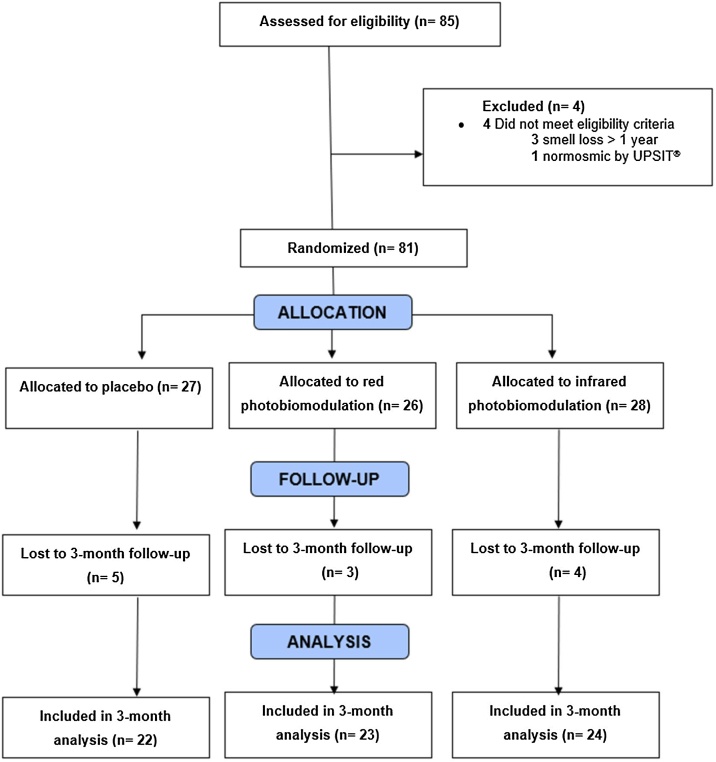
A flow diagram of study recruitment according to CONSORT guidelines. All patients received prednisolone 40 mg for seven days since the beginning of photobiomodulation and olfactory training with rose, lemon, clove, and eucalyptus essences twice a day throughout the study period.

Eighty-one patients 19–59 years of age who experienced persistent olfactory disturbances 1–12 months after SARS-CoV-2 infection were recruited by personal and social media advertisement between January 2021 and October 2022. Recruitment flow diagram is depicted in Fig. 1 according to CONSORT guidelines. Patients with preexisting smell alterations, a history of traumatic brain injury with loss of smell, neurological disorders, classification of normosmia in the first University of Pennsylvania Smell Identification Test (UPSIT®) assessment,^29^ and the impossibility of completing the proposed follow-up period were excluded.

### Outcomes

All study patients had their olfactory function assessed using the UPSIT®. This test comprises four booklets of 10 pages, each containing microencapsulated odor strips. The test score is determined by the number of odors correctly identified and classifies olfactory function as follows. For men, normosmia corresponds to a score between 32 and 40, hyposmia is divided into mild (28–31), moderate (24–27), and severe (17–23), and anosmia is defined as 16 or lower. For women, normosmia corresponds to a score between 35 and 40, mild hyposmia ranges from 31 to 34, moderate hyposmia from 26 to 30, and severe hyposmia from 19 to 25, while anosmia is defined as a score of 18 or lower. The primary outcome was the change in the UPSIT® score from baseline. In addition to the UPSIT®, secondary outcomes included subjects rating their self-perceived smell and taste abilities on a 10-point scale, with 0 being a total lack of sensation and 10 normal sensations. We also measured and compared the response rate in each group, defined as a 4 or more points increase in UPSIT® scores.

### Study design

**Fig. 2:**
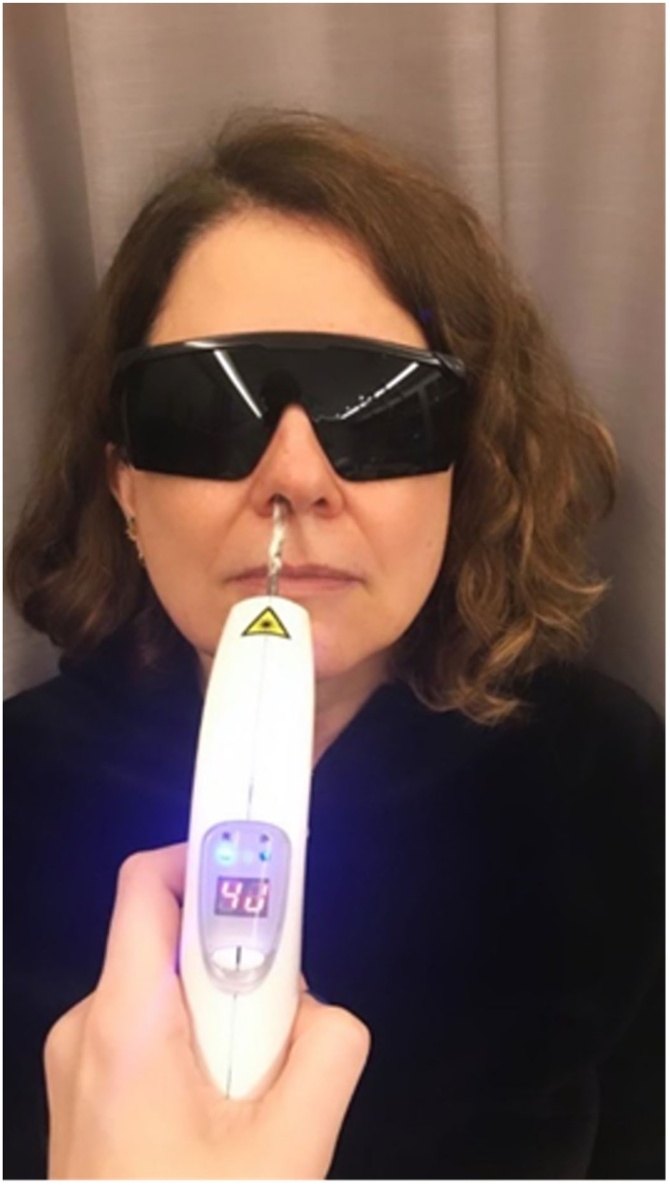
A subject is shown performing photobiomodulation, positioned seated with the head slightly tilted back. The applicator tip was inserted into the nostrils at around 1 cm for 40 seconds (video available online).

Patients were randomized into three groups (1:1:1) using a permuted block design with a block size of 6. This ensured a balanced distribution of interventions among the groups. The random allocation sequence was generated using an online random number generator service (Random.org) and implemented through sequentially numbered containers. To conceal the sequence until interventions were assigned, the containers were stored in a secure location, accessible only to the study coordinator, who was responsible for conducting the allocation. The study coordinator generated the random allocation sequence, enrolled participants, and assigned participants to interventions, ensuring the process was conducted in a blinded manner for both evaluators and participants. Controls underwent ten sessions of photobiomodulation therapy with the device turned on but without emitting any light, for 40 seconds in each nostril, twice a week. The red-light group was also submitted to 10 sessions with the device applying 4 J of red light in each nostril for 40 seconds twice a week, with an interval of 2–3 days (Fig. 2). In the third group, treated with infrared PBMT, ten sessions of PBMT were performed with the same number of sessions and frequency. All subjects wore PBMT light protection glasses which also blinded them to the applied therapy.

The recruited patients underwent olfactory training, smelling four different odors ‒ phenyl ethyl alcohol, eugenol, citronellal, and eucalyptol ‒ for 10 seconds, twice a day, for 90 days.^30^ In addition, they took 40 mg of prednisolone for seven days beginning on the first day of PBMT.

PBMT was done using the EC Therapy device (DMC, São Paulo, SP), a low-intensity laser (100 mW), with red and near-infrared light, with a wavelength of 660 nm and 780 nm, respectively. The dosimetric parameters were 4 J applied intranasally for 40 seconds inside each nostril and an energy density of 140 J/cm^2^. Laser protection goggles were mandatory for both the patient and the professional during the application. The device probe was disinfected and covered with disposable covers before intranasal use, following the manufacturer's recommendations and in compliance with the safety standards established by the National Health Surveillance Agency (ANVISA) for low-intensity laser equipment.

After 90 days of treatment, the study patients repeated the UPSIT® and the subjective assessments.

### Statisical analysis

We described continuous variables as means and standard deviations and categorical variables as percentages. We consider a 4-point difference between groups after PBMT to be a Minimum Clinically Important Difference (MCID).^31^, ^32^ This value was used to calculate the sample size, with an alpha level of 5%, a power of 80%, and a standard deviation of 4 points. The required sample determined for each group was 17 and was increased to 21, considering an attrition rate of 20%.

After verification of normality using the Shapiro–Wilk test, continuous variables were compared between groups or using the ANOVA or Kruskal-Wallis test and correlated with the Pearson’s test (*r*). Categorical variables are presented in number and percentage and compared using Fisher's exact test. Random effect mixed models with repeated measures, with the factor of the time of the visit or logistic regressions models were employed to compare changes or response rates between groups from baseline to 3-month follow-up visits, controlling for sex, ethnicity, olfactory loss time, and baseline UPSIT® or baseline subjective scores. A p-value lower than 0.05 was considered statistically significant. Statistical analysis was performed using Stata (version 13; Stata Corp., Texas, USA) and in R (The R Foundation for Statistical Computing, 2020) via RStudio.

## Results

Eighty-five patients were evaluated for eligibility. Three were excluded for participation for having smell loss for more than one year after their acute infection and one for exhibiting normosmia on UPSIT®. The remaining 81 enrolled patients were allocated to control (n = 27), red light (n = 26), and infrared light (n = 28) groups. In the control, red, and infrared groups, five, three, and four patients, respectively, did not complete all sessions and were excluded from the analysis. These 12 patients failed to respond to the follow-up at the 3-month mark. While some cited a lack of time as the reason for their withdrawal, others did not provide any response. Consequently, they were excluded from the study due to non-compliance with the study protocol. Clinical and demographic characteristics of the participants can be seen in Table 1. Age and baseline olfactory scores were similar between groups, and a slightly better self-reported taste capacity was found in patients undergoing infrared therapy (*p* = 0.010). The group using infrared had a lower proportion of women (*p* = 0.030) and controls had more non-white ethnicity (*p* = 0.030).

**Table 1:** Demographic and clinical characteristics of the patients.

Characteristics	Total (n = 81)	Controls (n = 27)	Red light (n = 26)	Infrared light (n = 28)	p-value
Age, mean (SD), y	36.2 (11.8)	36 (10.5)	36.2 (12.4)	36.4 (12.7)	0.990
Sex, n (%)					**0.030**
Female	58 (71.6)	23 (85.2)	20 (76.9)	15 (53.6)	
Male	23 (28.4)	4 (14.8)	6 (23.1)	13 (43.4)	
Race, n (%)					**0.030**
White	37 (45.7)	8 (29.6)	17 (65.4)	12 (48.9)	
Non-white	44 (54.3)	19 (70.4)	9 (34.6)	16 (57.1)	
Smoking, n (%)					0.550
Smoker or former smoker	23 (28.4)	8 (29.6)	9 (34.6)	6 (21.4)	
Non-smoker	58 (71.6)	19 (70.4)	17 (65.4)	22 (78.6)	
Comorbidities, n (%)					0.520
Yes	17 (21)	6 (22.2)	7 (26.9)	4 (14.3)	
No	64 (79)	21 (77.8)	19 (73.1)	24 (85.7)	
Hypertension	5 (6)	1 (3.7)	2 (7.7)	2 (7.1)	0.870
Diabetes	2 (2.4)	1 (3.7)	0	1 (3.6)	1
Asma	1 (1.2)	0	1 (3.8)	0	1
Depression	7 (7.4)	3 (11.1)	3 (11.5)	0	0.180
Education Level					0.147
Bachelors or Higher	48 (59.3)	15 (55.6)	20 (76.9)	13 (46.4)	
Middle School	26 (32.1)	9 (33.3)	4 (15.4)	13 (46.4)	
High School	7 (8.6)	3 (11.1)	2 (7.7)	2 (7.2)	
Olfactory loss time, mean (SD), months	7.2 (3.6)	7.8 (2.9)	6 (3.9)	7.9 (3.7)	0.100
Baseline UPSIT® score, mean (SD)	19.8 (7.4)	18.7 (7.3)	21.6 (6.5)	18.9 (8.2)	0.290
Baseline subjective olfactory score^a^	2.5 (1.7)	2.2 (1.5)	2.6 (2)	2.7 (1.8)	0.560
Baseline subjective taste score^a^	4.6 (2.9)	3.4 (2.3)	4.6 (2.7)	5.8 (3.2)	**0.010**

a Self-perceived smell and taste abilities on a 10-point scale, with 0 being a total lack of sensation and 10 normal sensations. Significant p-values are bolded.

**Fig. 3:**
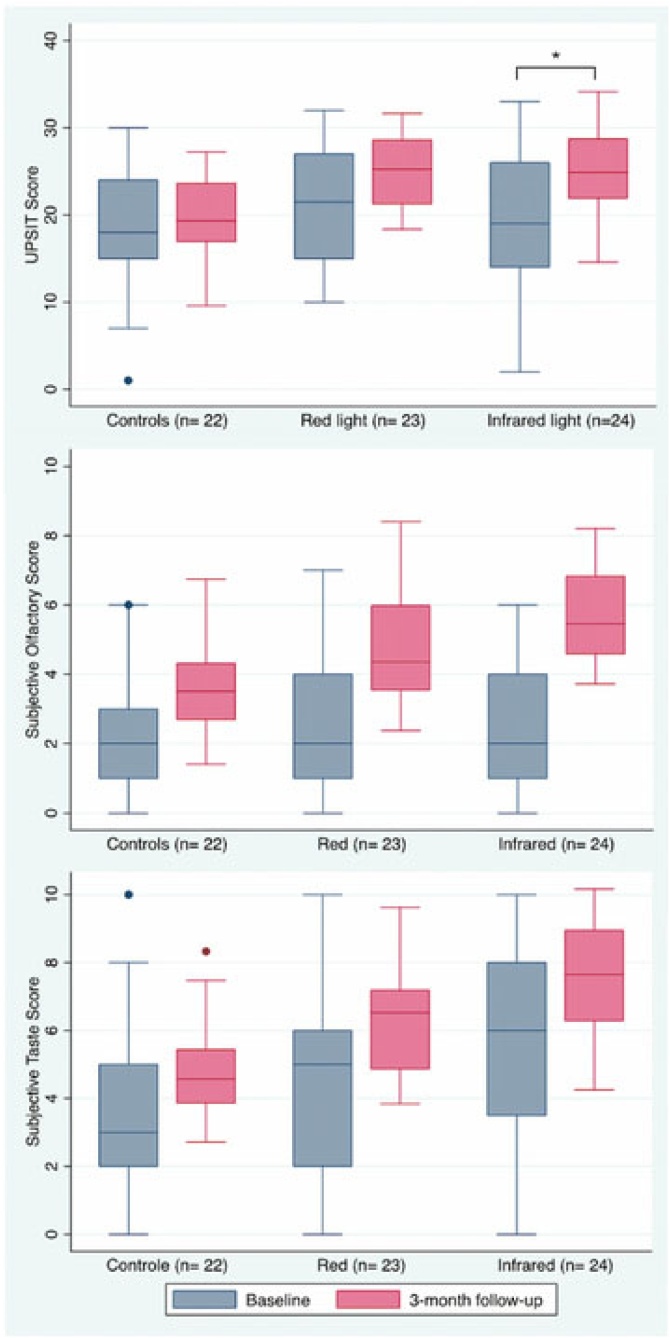
Comparison of UPSIT® scores, self-reported olfactory and taste capacities before and after treatment for the three study groups. The 3-month measurements are adjusted for baseline scores, sex, and ethnicity. Edges of boxes reflect the interquartile range. Sample sizes are in parentheses. Self-reported smell and taste capacities are on a 10-point scale, with 0 noting a total lack of sensation and 10 normal sensation. The asterisk denotes a statistically significant difference in the mixed model.

The effects of the PBMT compared to controls are depicted in Table 2 and Fig. 3. Compared to controls at the third month visit, patients undergoing infrared therapy achieved a statistically significant improvement in UPSIT® (*p* = 0.004) and subjective smell score (*p* = 0.009), in addition to a tendency toward an increased subjective ability to smell and taste (respectively *p* = 0.080 and *p* = 0.060). Considering only patients with 6 or more months of olfactory loss, this benefit of the infrared therapy persisted (coefficient of +6.6 (95% CI [3.4–9.9], *p* < 0.001).

**Table 2:** UPSIT® and self-reported scores before and at the 3-month follow-up visit following the initiation of the photobiomodulation therapy.

UPSIT® scores, mean (SD)	Baseline	3-month	Change^b^ (95% CI)	p-value
Controls	18.7 (7.3)	20.3 (7.4)	reference	Reference
Red light	21.6 (6.5)	25.3 (7)	+ 2 (−1.3–5.1)	0.230
Infrared light	18.9 (8.2)	25.3 (6)	+ 4.6 (1.5–7.8)	**0.004**

Subjective olfactory score^a^, mean (SD)	Baseline	3-month	Change (95% CI)	p-value
Controls	2.2 (1.5)	3.6 (3)	reference	Reference
Red light	2.6 (2)	4.4 (2.5)	+ 0.2 (−1.2 – 1.5)	0.200
Infrared light	2.7 (1.8)	5.7 (1.8)	+ 1.2 (−0.2–2.5)	0.080

Subjective taste score^a^, mean (SD)	Baseline	3-month	Change (95% CI)	p-value
Controls	3.4 (2.3)	4.5 (2.9)	reference	Reference
Red light	4.6 (2.7)	6.3 (2.7)	+ 0.8 (−0.4 – 2.8)	0.310
Infrared light	5.8 (3.2)	7.2 (2.5)	+ 1.3 (−0.23 – 3.3)	0.110

a Self-perceived smell and taste abilities on a 10-point scale, with 0 being a total lack of sensation and 10 normal sensations. Significant *p* values are bolded.

b These values represent the coefficients of random effect mixed models with repeated measures controlling for sex, ethnicity, time of olfactory dysfunction onset and visit.

Response rates were 26.1% (95% CI 6.7–45.5), 43.5% (95% CI: 21.5–65.4), and 68% (95% CI: 48.3–87.7) in control, red, and infrared groups, respectively. The infrared group had 8.3 times more likely to respond than controls (*Odds Ratio*, 95% CI: 1.8–38.1, *p* = 0.007). Forty-six and 37.5 percent of the patients returned to normosmia levels after red and infrared PBMT, respectively, while none of the controls recovered to this smell capacity (Table 3).

**Table 3:** Olfactory function according to UPSIT® before and at 3-month follow-up divided by groups.

Controls n (%)			
Before		After	
Anosmia	10 (45.5)	Anosmia	3 (30)
		Hyposmia	7 (70)
		Normosmia	0
Hyposmia	12 (54.5)	Anosmia	1 (8.3)
		Hyposmia	10 (83.4)
		Normosmia	1 (8.3)

Red light n (%)			
Before		After	
Anosmia	10 (43.5)	Anosmia	2 (20)
		Hyposmia	8 (80)
		Normosmia	0
Hyposmia	13 (56.5)	Anosmia	0
		Hyposmia	7 (53.8)
		Normosmia	6 (46.2)

Infrared light n (%)			
Before		After	
Anosmia	8 (33.3)	Anosmia	1 (12.5)
		Hyposmia	7 (87.5)
		Normosmia	0
Hyposmia	16 (66.7)	Anosmia	0
		Hyposmia	10 (62.5)
		Normosmia	6 (37.5)

Considering the prognostic factors with the three treatments, the lower the initial UPSIT® score, the greater the chance of having a Clinically Minimally Important Difference (MCID) after three months, with four or more points of improvement in this test (OR = 0.86 (95% CI [0.79, 0.95], *p* = 0.003). The Pearson correlation coefficient (*r*) between initial UPSIT® scores and score difference after the interventions was observed to be −0.46, indicating a moderate negative correlation (*p* < 0.001). Also, in all groups, age, sex, ethnicity, and time of onset of olfactory loss did not interfere with this better result (*p* > 0.050). When dividing the patients into the three treatment groups, only the infrared group-maintained significance in terms of better efficacy with a lower initial UPSIT score (*p* = 0.030), while age, gender, ethnicity, and time of olfactory loss continued to be unrelated to the best result (*p* > 0.050).

One patient reported a burning sensation during the application of PBMT, and another reported headache during the treatment, which may also be related to the associated olfactory training during the trial. Patients reported no other adverse events.

## Discussion

This study presents evidence regarding the efficacy of Photobiomodulation Therapy (PBMT) in the treatment of olfactory disturbances following SARS-CoV-2 infection. The findings have implications for the management of post-viral olfactory dysfunction and warrant further attention in the field of olfactory disturbances. This single-blinded, randomized, controlled clinical trial found significant olfactory improvement in patients receiving infrared PBMT compared to the control group. Self-perceived smell and taste abilities further supported the positive impact of infrared PBMT on this post-viral olfactory dysfunction.

A finding of this study was the association between lower initial UPSIT® scores and a higher likelihood of clinically significant improvement after three months of treatment. This suggests that patients with more severe olfactory dysfunction at baseline may experience greater benefit from PBMT.^20^ Patients with higher initial scores may still derive benefits from PBMT. However, the ceiling effect in obtaining initial higher scores might hinder the detection of subtle advantages. This gain could be observed in qualitative smell dysfunctions like parosmia and phantosmia, which were not the focal points of this study. However, other factors, such as age, sex, ethnicity, and time of onset of olfactory loss, did not influence treatment outcomes significantly.^16^, ^30^, ^32^ These findings highlight the potential for infrared PBMT to provide effective treatment irrespective of demographic or clinical characteristics.^25^, ^34^ Our study population primarily consists of individuals who are relatively young. Thus, we were unable to effectively evaluate the impact of laser therapy on individuals aged 60 and above.^26^

None of the anosmic individuals achieved normosmia, which is the ultimate goal of any therapy, highlighting the longer recovery time and increased treatment requirements for these patients.^6^, ^15^, ^31^ However, within the red and infrared PBMT groups, a significant number of others reached normosmia, which is a highly encouraging outcome. Specifically, 46% and 37.5% of the patients in the red and infrared groups, respectively, regained their sense of smell to normal levels. Notably, none of the control subjects were able to recover their olfactory capacity to the same extent.^15^ It is essential to consider that some patients were already near the normal range at the baseline, so achieving normosmia through the olfactory test may not necessarily indicate a dramatic treatment effect.

The safety profile of PBMT was well-established throughout the study. Only two minor adverse events were reported, including a burning sensation and headache, both of which were transient and not directly attributed to the therapy. This favorable safety profile adds to the feasibility and acceptability of PBMT as a treatment modality for post-viral olfactory dysfunction.^24^, ^26^

The mechanisms underlying the therapeutic effects of PBMT on olfactory function remain unclear. Further research is warranted to elucidate the specific biological processes involved.^33^ Additionally, long-term follow-up studies would provide valuable insights into the durability of treatment effects and potential for sustained improvement beyond the three-month period evaluated in this study.^27^

The results of this study have important clinical implications for the management of patients with post-viral olfactory dysfunction. Current treatment options for this condition are limited, with limited evidence supporting their efficacy. The findings suggest that incorporating infrared PBMT, alongside olfactory training, into the treatment regimen may improve olfactory function and enhance patients’ quality of life.

It is important to acknowledge certain limitations of this study. First, the study focused specifically on patients with olfactory disturbances following SARS-CoV-2 infection and on a younger skewed population (maximum age was 59), limiting its generalizability to other causes of post-viral olfactory dysfunction and older patients. Second, a higher proportion of men was randomized for the infrared group and of non-white in the controls, two potential confounders for olfactory perception, controlled in the multivariate analysis employed. Secondly, a higher proportion of men were randomized into the infrared group, while the control group had a higher representation of non-white individuals. These demographics, known as potential confounders for olfactory perception, were controlled for in the employed multivariate analysis. Third, the study was not performed in a double-blind manner and no nasal endoscopy was performed before the laser application. Fourth, it is not known whether PBMT alone can produce the apparent changes.

Future research should explore the applicability of PBMT in diverse patient populations, including those with olfactory dysfunction of non-viral etiology.

## Conclusions

This study provides preliminary evidence of the efficacy of infrared PBMT in treating post-viral olfactory dysfunction. The findings underscore the potential of PBMT as a novel therapeutic option, offering hope to patients experiencing olfactory disturbances following SARS-CoV-2 infection. Further research is warranted to expand our understanding of the underlying mechanisms and optimize treatment protocols. Continued investigation in this area may pave the way for improved management strategies and better outcomes for patients with post-viral olfactory dysfunction.

## CRediT authorship contribution statement

Marco Aurelio Fornazieri: Conceptualized and designed the study, conducted the data collection, conducted the analyses, drafted the initial manuscript, and revised the manuscript.

Patricia Costa Oliveira and Luisa Oliveira Correia: Conceptualized and designed the study, conducted the data collection, drafted the initial manuscript, and reviewed the manuscript.

Gabriel Rodrigues Suassuna: Helped with data collection and reviewing the manuscript.

Natalia Medeiros Dias Lopes, Fabio de Rezende Pinna, Richard Louis Voegels, and Richard L Doty: Provided statistical guidance and critically revised the manuscript. All authors approved the final manuscript as submitted.

## Justification of the number of authors

The number of authors was essential considering the pandemic and the need for several people to carry out the tests and recruitment.

## Disclosures

No authors have disclosures.

## Funding source

None

## Declaration of competing interest

The authors declare no conflicts of interest.

## Appendix A Supplementary data

Video The video demonstrates the process of connecting the equipment and highlights the protocols employed. To begin, the equipment is powered on using the red wavelength along with the 4 joules protocol. It is then placed inside the nostril, where the red light becomes visible. Subsequently, we present an alternate protocol that solely varies in terms of wavelength, utilizing infrared light which is not visible to the naked eye.
