# Supplementary material for: Efficacy of the adjunctive use of photobiomodulation therapy in olfactory disorders in post-COVID-19 patients: A randomized controlled trial
Source: Braz J Otorhinolaryngol. 2025 Mar 26;91(4):101583. doi: 10.1016/j.bjorl.2025.101583 (PMC11986229; doi:10.1016/j.bjorl.2025.101583)
Supplement: Video — The video demonstrates the process of connecting the equipment and highlights the protocols employed. To begin, the equipment is powered on using the red wavelength along with the 4 joules protocol. It is then placed inside the nostril, where the red light becomes visible. Subsequently, we present an alternate protocol that solely varies in terms of wavelength, utilizing infrared light which is not visible to the naked eye. [file mmc1.docx]

BJORL-D-24-00394_Supplementary Material

**Video** The video demonstrates the process of connecting the equipment and highlights the protocols employed. To begin, the equipment is powered on using the red wavelength along with the 4 joules protocol. It is then placed inside the nostril, where the red light becomes visible. Subsequently, we present an alternate protocol that solely varies in terms of wavelength, utilizing infrared light which is not visible to the naked eye.
